# A novel gene expression signature-based on B-cell proportion to predict prognosis of patients with lung adenocarcinoma

**DOI:** 10.1186/s12885-021-08805-5

**Published:** 2021-10-12

**Authors:** Yi Zhang, Xuewen Yin, Qi Wang, Xuming Song, Wenjie Xia, Qixing Mao, Bing Chen, Yingkuan Liang, Te Zhang, Lin Xu, Feng Jiang, Xinyu Xu, Gaochao Dong

**Affiliations:** 1 Department of Thoracic Surgery, Jiangsu Cancer Hospital, Jiangsu Institute of Cancer Research, The Affiliated Cancer Hospital of Nanjing Medical University, 210000 Nanjing, P. R. China; 2 Department of Pathology, Jiangsu Cancer Hospital, Jiangsu Institute of Cancer Research, The Affiliated Cancer Hospital of Nanjing Medical University, 210000 Nanjing, P. R. China; 3 Jiangsu Key Laboratory of Molecular and Translational Cancer Research, Cancer Institute of Jiangsu Province, Nanjing, P. R. China; 4 School of Basic Medicine and Clinical Pharmacy, China Pharmaceutical University, 211198 Nanjing, P. R. China; 5 Department of Radiation Oncology, Jiangsu Cancer Hospital, Jiangsu Institute of Cancer Research, The Affiliated Cancer Hospital of Nanjing Medical University, 210000 Nanjing, P. R. China; 6 The Fourth Clinical College of Nanjing Medical University, Nanjing, P. R. China

**Keywords:** B cells, Lung adenocarcinoma, Microenvironment, Prognosis model, Immunotherapy management

## Abstract

**Background:**

This study aimed to develop a reliable immune signature based on B-cell proportion to predict the prognosis and benefit of immunotherapy in LUAD.

**Methods:**

The proportion of immune cells in the TCGA-LUAD dataset was estimated using MCP-counter. The Least Absolute Shrinkage and Selector Operation was used to identify a prognostic signature and validated in an independent cohort. We used quantitative reverse transcription-polymerase chain reaction (qRT-PCR) data and formalin-fixed paraffin-embedded (FFPE) specimens immunohistochemistry to illustrate the correlation between prognostic signature and leukocyte migration.

**Results:**

We found that the relative abundance of B lineage positively correlated with overall survival. Then, we identified a 13-gene risk-score prognostic signature based on B lineage abundance in the testing cohort and validated it in a cohort from the GEO dataset. This model remained strongly predictive of prognoses across clinical subgroups. Further analysis revealed that patients with a low-risk score were characterized by B-cell activation and leukocyte migration, which was also confirmed in FFPE specimens by qRT-PCR and immunohistochemistry. Finally, this immune signature was an independent prognostic factor in the composite nomogram of clinical characteristics.

**Conclusions:**

In conclusion, the 13-gene immune signature based on B-cell proportion may serve as a powerful prognostic tool in LUAD.

**Supplementary Information:**

The online version contains supplementary material available at 10.1186/s12885-021-08805-5.

## Introduction

Lung cancer is one of the malignant tumors with the highest morbidity and mortality worldwide; the overall 5-year survival rate is about 20% [1]. Nonsmall cell lung cancers (NSCLCs) represent 85% of lung tumors. They encompass multiple cancer types, such as lung adenocarcinomas (LUADs), lung squamous cell carcinomas (LUSCs), and large-cell cancers. Among them, LUADs and LUSCs are the largest NSCLC subgroups [2]. Therefore, effective treatments for LUAD have always been the focus of research. Over the past 10 years, the understanding of the immune system and its role in the development and progression of cancer has continued to deepen, leading to remarkable progress in the field of cancer immunotherapy [3]. Immunotherapy has been widely used in the first-line and second-line treatments of NSCLC [4–6], which has inspired people’s enthusiasm for elucidating the prognostic and pathophysiological effects of the tumor microenvironment (TME). The TME, including cancer-associated fibroblasts [7, 8], extracellular matrix [9], epithelial cells [10], myeloid cells [11], and tumor-infiltrating lymphocytes [12], affects the malignant progression and immune response of lung cancer. T cells and B cells are important components of tumor-infiltrating immune cells. The research on the functions and mechanisms of T cells is relatively comprehensive; however, the research on B cells is still insufficient.

Tumor-infiltrating B cells have emerged as key players in the TME. Chen and colleagues performed a single-cell RNA-seq analysis of cells isolated from patients with NSCLC and identified two major subtypes of B cells, namely the naïve-like and plasma-like B cells [13]. They found that the naïve-like B cells suppressed growth, while the plasma-like B cells promoted cell growth in the advanced stage of NSCLC, but inhibited cancer cell growth in the early stage of NSCLC. Wang and colleagues conducted a comprehensive genomic landscape of 149 NSCLC cases and revealed that highly clustered *EGFR* mutations were associated with inflammatory tumor-infiltrating B lymphocytes, which was also confirmed in the TCGA dataset [14]. Tumor-infiltrating B cells also served as local antigen-presenting cells by providing secondary stimulation to Immune infiltrating cells (TILs). Bruno and colleagues demonstrated that tumor-infiltrating B cells efficiently presented antigens to CD4^+^ TILs and identified three CD4^+^ TIL responses to tumor-infiltrating B cells, which were categorized as activated, antigen-associated, and nonresponsive [15]. Hence, a new role was suggested for tumor-infiltrating B cells in their interplay with CD4^+^ TIL in the TME. Whether tumor-infiltrating B cells have protumor or antitumor effect is still controversial.

Considering the important roles of B cells in the TME, which constitutes a potential novel therapeutic in NSCLC immunotherapy, urged us to construct a comprehensive approach to identify various charatacteristics of LUAD including B cell function, patients outcome and immunotherapy benefits. Therefore, a prognosis signature based on B-cell proportion was established, which was a robust prognostic biomarker and predictive factor that could be used in the clinic.

## Materials and methods

### RNA-sequencing data used to assess the abundance of immune-infiltrating cells

The gene expression data (workflow type: HTSeq-Counts) and the corresponding clinical information from the Cancer Genome Atlas (TCGA) website (https://gdc.cancer.gov/) were downloaded using the “TCGAbiolinks” R package (Version 2.14.1). Entrez IDs were converted into gene symbols using the Bioconductor package “org. Hs.eg.db” (Version 3.10.0). Genes with low expression were removed from the profile. The abundance of immune-infiltrating cells in each sample was assessed with the MCP-counter [16], which provided the abundance score for eight immune populations (T cells, CD8+ T cells, cytotoxic lymphocytes, natural killer cells, B lineages, monocytic lineage, myeloid dendritic cells, and neutrophils) and two stromal populations (endothelial cells and fibroblasts). The assessment of these cell subpopulations was based on the analysis of gene expression of cell markers. The MCP-counter signature composition of B lineages was as follows: *BANK1*, *CD19*, *CD22*, *CD79A*, *CR2*, *FCRL2*, *IGKC*, *MS4A1*, and *PAX5*. The transcripts of other cell subpopulations were published by the algorithm’s author. All cell subpopulation abundances were normalized using the *Z* score.

### Differential expression analysis and construction of the B-lineage-associated risk signature

Differential expression analysis between high B-lineage infiltration group and low B-lineage infiltration group was performed using the “DESeq2” R package (Version 1.26.0) with the standard comparison mode between the two experimental conditions. FoldChange > 3 and *P* value < 0.01 genes were selected for followup research. LASSO algorithm, using the R package “glmnet” (Version 3.0), was built to construct a B-lineage-associated risk signature. The “survival” R package (Version 3.5) was used to select the optimal cutoff value and plot Kaplan–Meier survival curves. The “timeROC” R package (Version 0.4) was used to conduct a time-dependent receiver operating characteristic (ROC) curve analysis.

### Microarray data

The transcript expression matrixes from GSE31908, GSE29013, and GSE30219 based on the GPL570 platform, including 131 patients with LUAD, were downloaded from the Gene Expression Omnibus (GEO) database. In these matrixes, the gene expression data for three matrixes were subjected to log2 transformation. The scale method of the “limma” R package (Version 3.42.2) was used to normalize the data.

### Patients with LUAD from Cancer hospital affiliated to Nanjing Medical University

A total of 12 patients who underwent surgery without neoadjuvant chemotherapy and were diagnosed with LUAD at Cancer Hospital Affiliated to Nanjing Medical University (Nanjing, China) were included. The Nanjing cohort consisted of formalin-fixed paraffin-embedded (FFPE) specimens collected from patients who underwent radical surgery between 2018 and 2020. Each patient underwent a standard radical surgical procedure, and all specimens were evaluated by expert pathologists according to eighth edition of the Union for International Cancer Control Tumor-Node-Metastasis (TNM) grading system. All patients underwent regional lymphadenectomy, and the existence of Tumor-Lymph Node-Metastasis (TNM) was pathologically examined. Total RNA was extracted from 4-μm-thick FFPE specimens by manual microdissection using an RNeasy FFPE Kit (Qiagen, Hilden, Germany). The complementary DNA (cDNA) synthesis was performed using PrimeScript RT Master Mix (RR036A) (Takara, Dalian, China). The quantitative reverse transcription–polymerase chain reaction (qRT-PCR) assays were performed with a ViiA 7 Dx RT-PCR System (Applied Biosystems, Foster City,USA) using PowerUp SYBR Green Master Mix (Applied Biosystems, Vilnius, Lithyania). The cycling conditions were as follows: 40 cycles of 95 °C for 15 s and 60 °C for 60 s. The relative expression of target genes was normalized against glyceraldehyde-3-phosphate dehydrogenase using the 2^–ΔCT^ method. Primer sequences are provided in Table S1.

### Immunohistochemistry

LUAD tissues were fixed with 10% formalin and embedded in paraffin. Then, the tissues were cut into 5-μm-thick sections and incubated overnight with primary antibodies anti-CD3, anti-CD4, anti-CD8, anti-CD19, anti-CD20, anti-PD1 (Abcam,UK). The sections were subsequently incubated with a secondary antibody (Abcam,UK) at 37 °C for 1.5 h and stained with a 3,3-diaminobenzidine solution.

### Function enrichment and gene interaction analyses

Gene Ontology and Kyoto Encyclopedia of Genes and Genomes analyses were performed using “clusterProfiler” R package (Version 3.11) based on differentially expressed genes (absolute value of logFC > 1.5; *P* value < 0.01). GeneMANIA (https://genemania.org/) was used to find other genes related to a set of input genes using a very large set of functional association data. Association data included protein and genetic interactions, pathways, co-expression, co-localization, and protein domain similarity.

### Development and validation of the nomogram

Univariate and multivariate Cox analyses were performed to assess the independent prognostic ability of B-lineage-associated risk signature using “survival” R package (Version 3.5). Then, a concise nomogram of predicting the OS of LUAD was established using R package “rms” (Version 2.10), including four factors. In addition, the predictive accuracies of the nomogram and separate prognostic factors were compared using ROC analyses.

### Statistical analysis

Statistical analyses were performed using R (Version 3.6.3) and GraphPad Prism 8. The Wilcoxon rank-sum test and Student *t* test were used to determine differences in comparison of two groups. All statistical tests were two-tailed with a statistical significance level set at 0.05 in this study.

## Results

### A landscape of immune infiltration of patients with LUAD

**Fig. 1:**
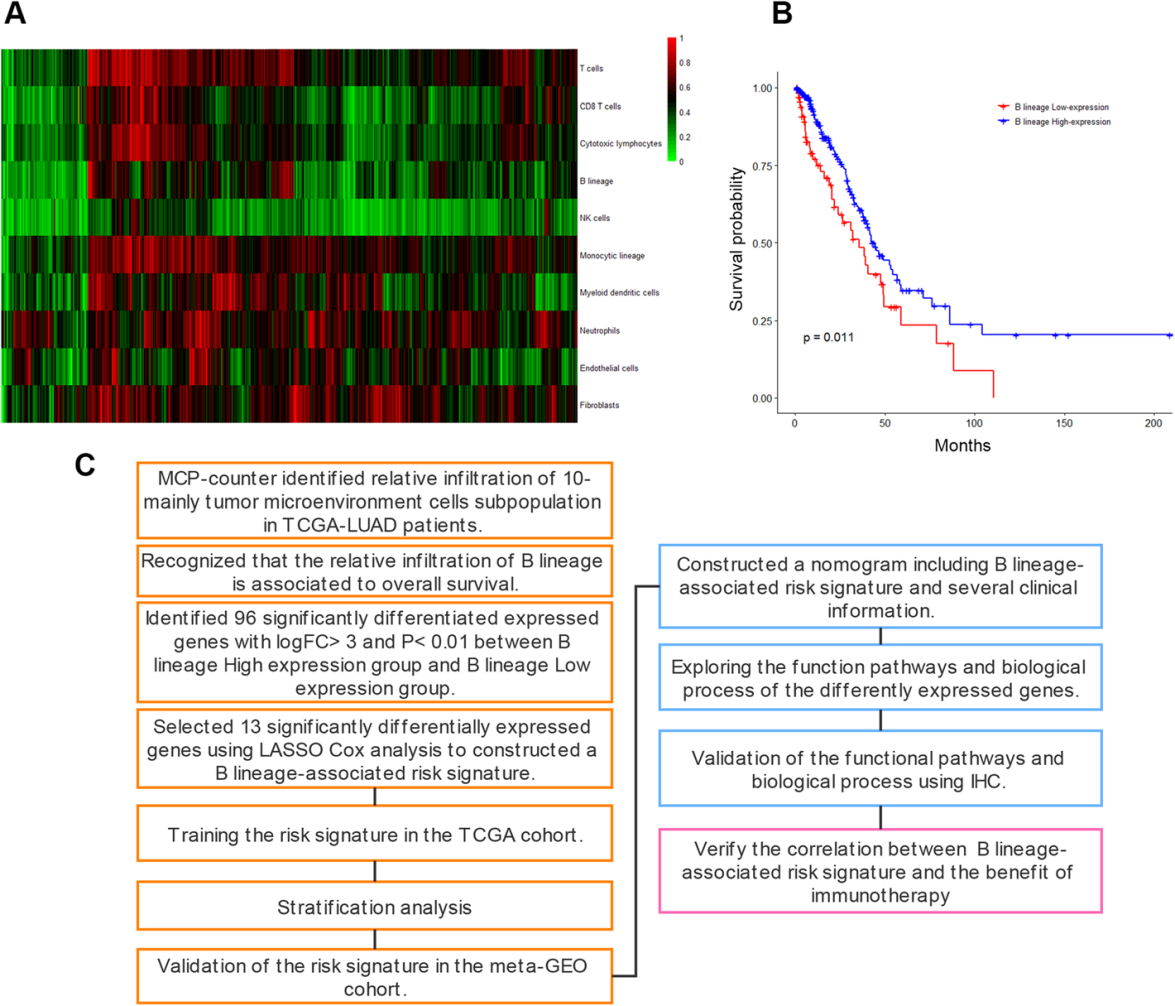
(**A**) A heatmap reflecting the relative abundance of immune microenvironment cell subpopulation in patients with lung adenocarcinoma in TCGA using MCP-counter R package. Expression profiles are normalized by z-score. (**B**) Kaplan-Meier survival curve analysis between high relative abundance of B lineage patients and low relative abundance B lineage patients. (**C**) The study design in this study

Immune cell infiltration and TME are vital in tumor immunity. In recent studies, MCP-counter was widely used to quantify the relative abundance of immune cell subpopulations through the expression of multiple immune-infiltrating cell markers, yielding robust results. A total of 400 patients from TCGA-LUAD were screened as TCGA cohort because of having relatively complete demographic information, clinical information, and survival information. MCP-counter was used to visualize the relative abundance of multiple immune-infiltrating cell subpopulations with Z score (Fig. 1A). The significant difference in the relative abundance of immune cell subpopulations between different samples was clearly observed. Among these immune cell subpopulations, patients were divided into low expression of B lineage (100 patients) and high expression of B lineage (300 patients). The relative abundance of B lineage significantly positively correlated with OS (Fig. 1B). However, MCP-counter was used to quantify the relative abundance of B lineage based on 400 samples in the TCGA cohort. It is difficult for individuals to quantify the abundance of B lineage. Therefore, a B-lineage-associated risk signature was constructed to assess the OS of patients with LUAD and guide individualized diagnosis and treatment strategies. The study design is shown in Fig. 1C.

### Construction of a B-lineage-associated risk signature

RNA expression profiles of 400 samples in the TCGA cohort were used to construct a B-lineage-associated risk signature predicting the OS of patients with LUAD; the gene expression of total RNA is shown in Fig. S1A. In the TCGA cohort, differently expressed genes were detected between low expression of B lineage (70 patients) and high expression of B lineage (330 patients), revealing that 1057 genes were statistically significantly differentially expressed, including 991 downregulated genes and 66 upregulated genes (Fig. S1B). Moreover, cluster analysis of these 1057 significantly differentially expressed genes in the TCGA cohort was performed (Table S1). A significant clustering was observed between the 2 groups of samples, confirming that these 1057 genes were statistically significantly differently expressed (Fig.S1C).

Furthermore, we filtered the differentially expressed genes with absolute value of logFC > 3 and *P* value < 0.01, including 95 downregulated genes and 1 upregulated genes, to identify genes with large differences between groups as B-lineage-associated risk signature candidate genes (Table S2). Further, 13 genes, including *FDCSP*, *FCER2*, *CNR2*, *MS4A1*, *FCRL1*, *BLK*, *TNFRSF13B*, *CD19*, *FCRLA*, *CR2*, *GH1*, *KRT20*, and *ALB*, with nonzero regression coefficients with 10-fold cross-validation were found to have maximum prognostic value according to LASSO Cox regression analysis (Fig. S1D and S1E). Finally, a 13-gene B-lineage-associated risk signature was constructed, and the risk score of this risk signature was calculated using the following formula:

$$ \mathrm{B}\;\mathrm{lineage}\hbox{-} \mathrm{associated}\kern0.17em \mathrm{risk}\kern0.17em \mathrm{signature}=\sum \limits_{\mathrm{i}=1}^{13}{Coeff}_i\ast {\left( Normalize\kern0.17em Expression\right)}_i $$

The coefficient of each gene is shown in Table S4. Based on the B-lineage-associated risk signature, patients were divided into two subgroups. The optimal cutoff value was determined using the “surv_cutpoint” function of the “survminer” R package; the optimal cutoff value was − 16.2 (Fig.S1F). The cutoff value in the TCGA cohort served as the cutoff value to assign patients into high-risk and low-risk groups across all patients with LUAD in the following analysis.

### Evaluation and validation of the prognostic ability of B-lineage-associated risk signature

**Fig. 2:**
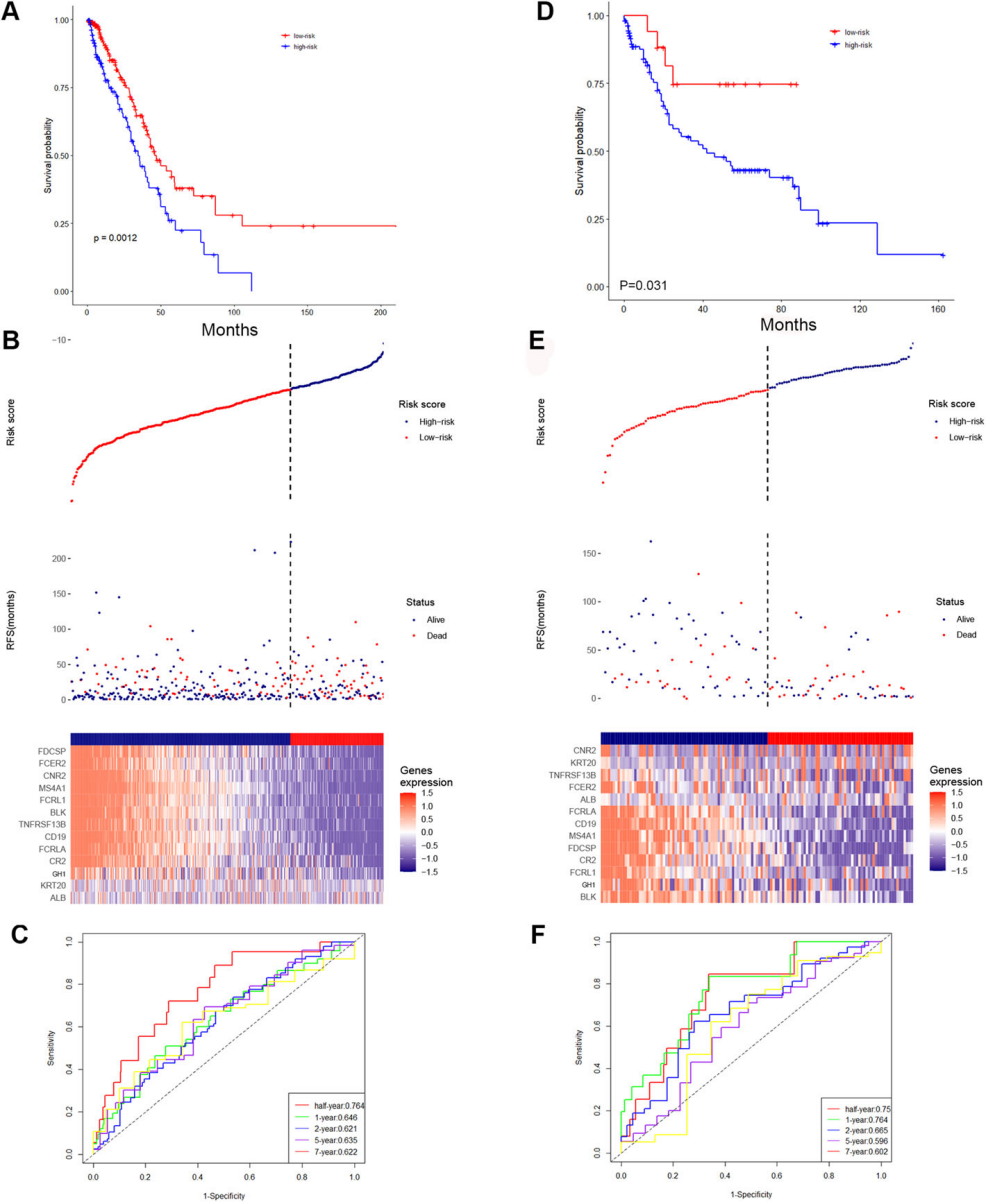
(**A**) The Kaplan-Meier survival analysis in the TCGA cohort. (**B**) The risk signature (upper), the OS (middle) and the expression of 13 selected genes (bottom) in the TCGA cohort. (**C**) The time-dependent ROC curves of TCGA cohort. (**D**) The Kaplan-Meier survival analysis in the meta-GEO cohort. (**E**) The risk signature (upper), the OS (middle) and the expression of 13 selected genes (bottom) in the meta-GEO cohort. (F) The time-dependent ROC curves of the meta-GEO cohort

The prognostic capability of the B-lineage-associated risk signature in the TCGA cohort was evaluated. Due to the limited patient records available, the duration of treatment is unknown. However, the diversity of treatment methods in patient samples reflects the challenging variables that clinicians encounter when treating LUAD. The Kaplan–Meier analysis demonstrated that the patients in the high-risk group correlated with worse outcome (Fig. 2A). The risk signature distribution, OS, and 13-gene expression profile are shown in Fig. 2B. In addition, the time-dependent ROC curve analysis of the of B-lineage-associated risk signature in the TCGA cohort revealed a promising prognostic capability for OS (half-year AUC = 0.764, 1-year AUC = 0.646, 2-year AUC = 0.621, 5-year AUC = 0.635, and 7-year AUC = 0.622, Fig. 2C).

The risk signature was assessed in an independent validation cohort, meta-GEO cohort, including GSE29013, GSE30219, and GSE31908, to validate the robust ability of the B-lineage-associated risk signature to predict OS. The cohort contained 131 patients with LUAD having survival information and RNA expression profiles. The Kaplan–Meier analysis yielded consistent results that patients in the high-risk group were positive related to worse clinical outcome (Fig. 2D). The risk signature distribution, OS, and 13-gene expression profile are shown in Fig. 2E. The time-dependent ROC curve analysis of the B-lineage-associated risk signature in the meta-GEO cohort validated the robustness of prognostic capability (half-year AUC = 0.75, 1-year AUC = 0.764, 2-year AUC = 0.665, 5-year AUC = 0.596, and 7-year AUC = 0.602, Fig. 2F). The aforementioned results indicated that the B-lineage-associated risk signature could predict OS.

### Stratification analysis of the B-lineage-associated risk signature

Multiple clinical factors have a significant impact on the survival of patients with LUAD. The stratification analysis of the B-lineage-associated risk signature in several clinical subgroups was performed to verify that the B-lineage-associated risk signature was independently involved in predicting prognosis, without being manipulated by any clinical subgroup. The Kaplan–Meier survival analysis between high-risk and low-risk groups in these subgroups illustrated that risk signature was still a robust and independent marker for predicting OS in patients with different sexes (Fig. S2A and S2B), different ages (Fig. S2C and S2D), and different TNM stages (Fig. S2E and S2F).

### Difference in immune status and immunotherapy benefits between high-risk and low-risk patients

Gene Ontology (GO) and Kyoto Encyclopedia of Genes and Genomes (KEGG) analyses of differently expressed genes were performed to further explore the pathways of statistically differently expressed genes between patients with low expression of B lineage and patients with high expression of B lineage and also understand the potential biological processes affecting the prognosis of patients (absolute value of logFC > 1.5, *P* value < 0.01, 1057 genes, Fig. S3A and S3B). The GO analysis comprised enriched biological processes, cellular components, and molecular function. These genes were enriched in several immune-related biological processes, including B-cell activation, leukocyte migration, humoral immune response, and regulation of B-cell receptor signaling pathway. In molecular function, these genes were enriched in immunoglobulin binding. The KEGG analysis reflected that these genes were enriched in cytokine–cytokine receptor interaction. These results indicated that differently expressed genes between patients with low expression of B lineage and patients with high expression of B lineage were related to the tumor immune process, suggesting that tumors evade immune surveillance and cause different clinical outcomes due to differences in immune cell activation in the TME and differences in antigen presentation and signal transduction,. Moreover, GeneMANIA online tool was used to observe the interaction of 13 risk signature–related genes (Fig. S3C). The result reflected that these genes were co-expressed and had close physical interactions. In addition, these genes had close relationship with several chemokine-related genes and B-cell surface marker–related genes. Moreover, we got consist result in the correlationship of 13 risk signature–related genes expression, which revealed a extensive co-regulation relationship (Fig. S3D). This finding suggested that these genes might have originated from the same underlying structure and might affect the B-cell activation and antigen presentation process, thereby affecting the prognosis of patients with LUAD.

**Fig. 3:**
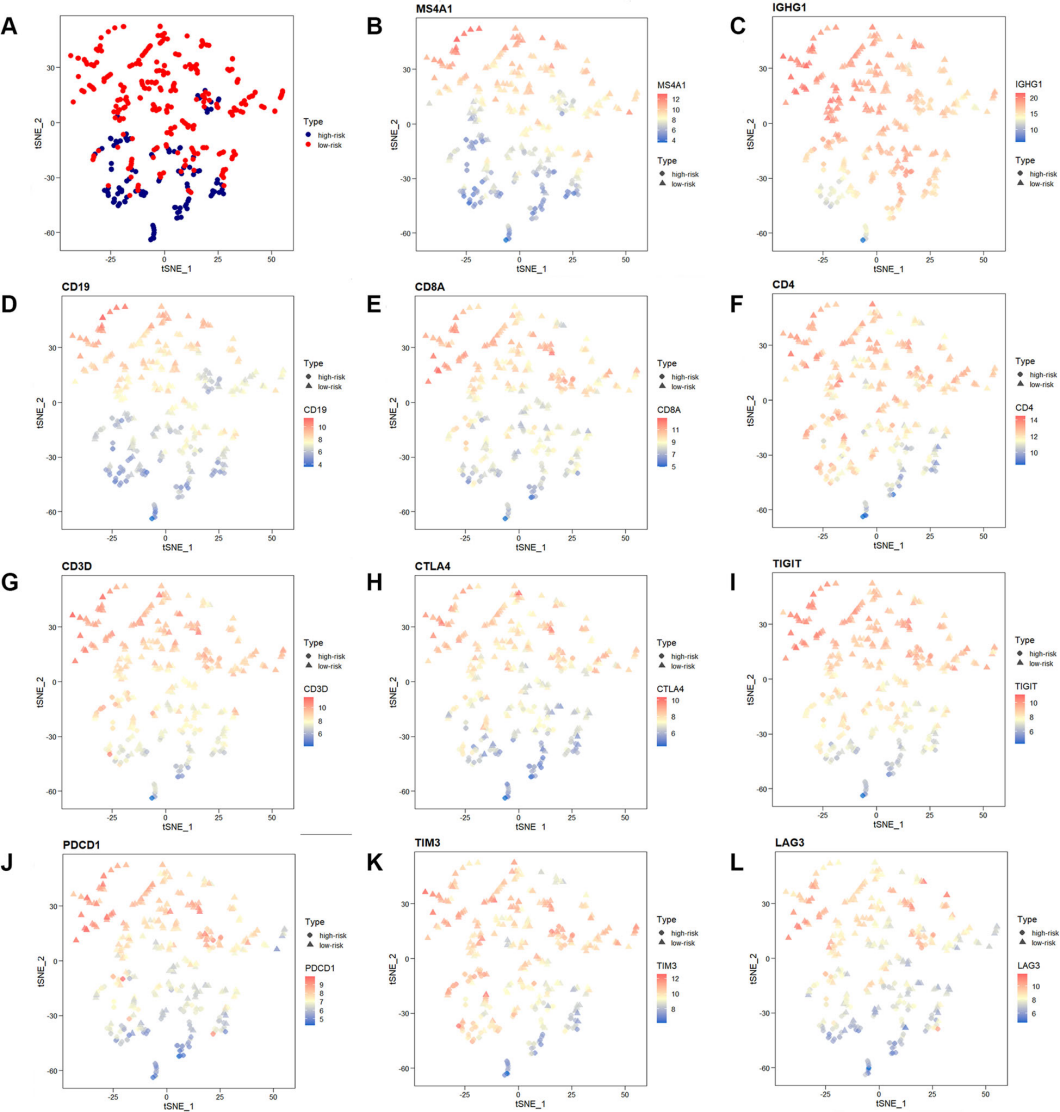
The expression difference of several immune cells markers including (**A**) MS4A1 (encoding CD20, marker of B cells), (**B**) IGHG1 (marker of plasma cells), (**C**) CD19 (encoding CD19, marker of B cells), (**D**) CD8A (encoding CD8, marker of CD8+ T cells), (**E**) CD4 (encoding CD4, marker of CD4+ T cells), (**F**) CD3D (encoding CD3, marker of T cells) and (**G**-**K**) expression of several immune checkpoints

Recent studies have shown that the difference between the TME and immune cell infiltration can significantly change the recognition of tumor antigens, presentation of signals, and killing of tumor cells by the patient immune system. The expression of immune cell markers was detected to examine the relative abundance of tumor suppressor immune cell subpopulations in the TME between high-risk and low-risk groups. The tSNE plot revealed that two groups of patients in the TCGA cohort were divided into two different principal component clusters based on mRNA expression (Fig. 3A). The expression levels of *MS4A1* (encoding CD20, marker of B cells, Fig. 3B), *IGHG1* (marker of plasma cells, Fig. 3C), and *CD19* (encoding CD19, marker of B cells, Fig. 3D) were significantly high in the low-risk groups, indicating higher abundance of the tumor suppressor B lineages. The expression levels of *CD8A* (encoding CD8, marker of CD8+ T cells, Fig. 3E), *CD4* (encoding CD4, marker of CD4+ T cells, Fig. 3F), and *CD3D* (encoding CD3, marker of T cells, Fig. 3G) were also significantly high in the low-risk groups, indicating higher abundance of the tumor suppressor T-cell subpopulation.

**Fig. 4:**
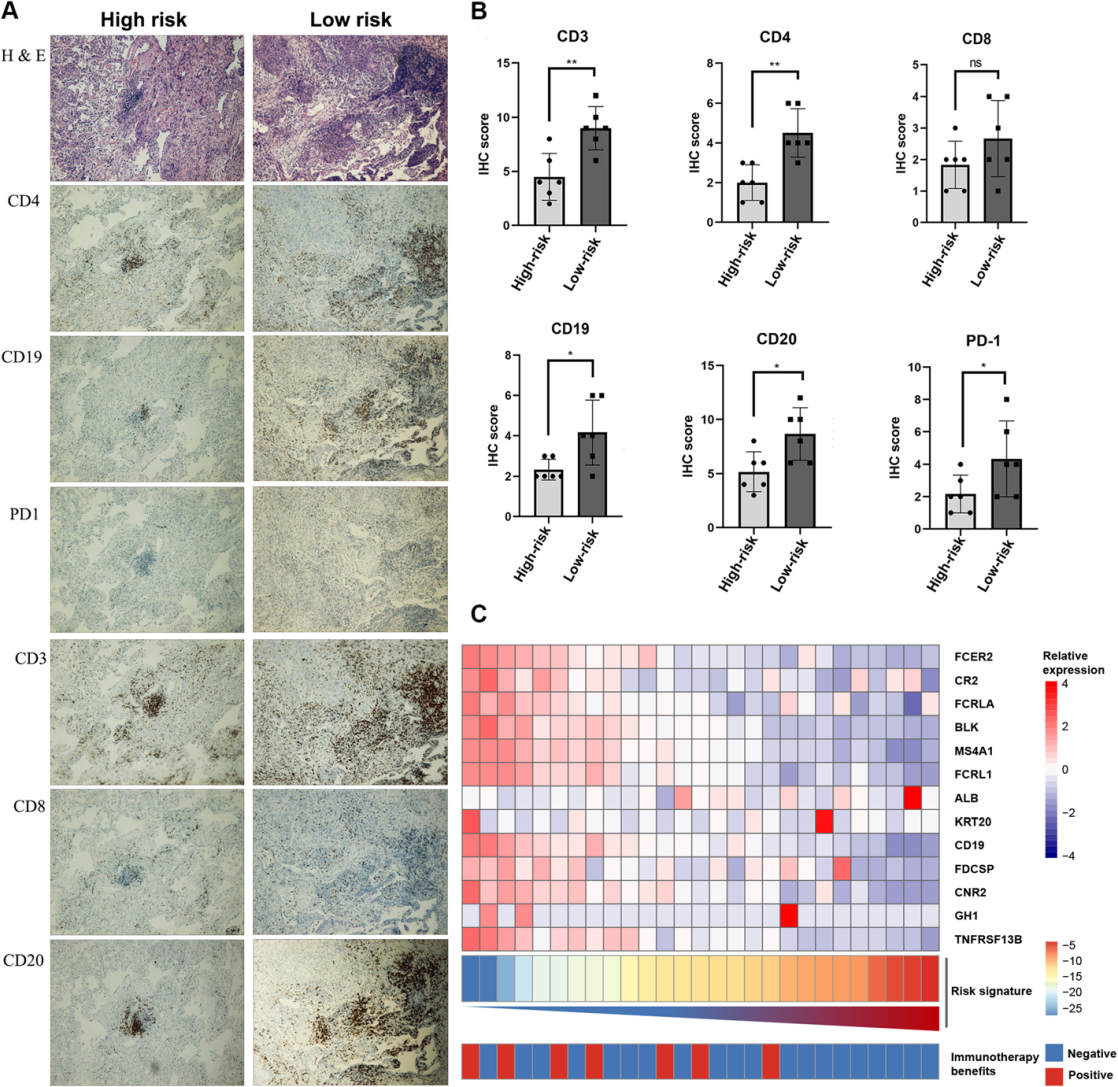
Immunohistochemical analysis of patients in high-risk group and low-risk group. (**A**) H&E representative pictures and immunohistochemical representative pictures of anti-CD3, anti-CD4, anti-CD8, anti-CD19, anti-CD20, and anti-PD1 in high-risk LUAD patients. (**B**) The IHC-score of those above antigens in low-risk group and high-risk group. (**C**) B-lineage-associated risk signature and immunotherapy benefit distribution heatmap

Meanwhile, immune checkpoints inhibitors have been shown to exert antitumor effects by reversing tumor-immunosuppressive effects. The present study further investigated the relationship between the B-lineage-associated risk signature and the expression of immune checkpoints, including *CTLA4*, *TIGIT*, *PDCD1*, *TIM3*, and *LAG3* (Fig. 3H–L). These results showed that these immune checkpoints were highly expressed in low-risk patients. In addition, patients were divided into high-risk and low-risk groups by qRT-PCR in 16 patients with LUAD from Jiangsu Cancer Hospital (The primers shown in Table S4). Immunohistochemical analysis was performed on the surface proteins of the immune cells and important immune checkpoints on paraffin-embedded pathological sections of these patients, including CD20, CD19, CD8, CD4, CD3, and PD1 (Fig. 4A and Table S5). Consistent results were obtained at the protein and RNA levels (Fig. 4B). Moreover, We continue to explore whether immunotherapy can benefit in the “immune activation” low-risk group. Unsurprisingly, B-lineage-associated risk signature and immunotherapy benefit showed a correlation trend (Fig. 4C) in GSE135222. These results indicated that high-risk and low-risk groups distinguished by the B-lineage-associated risk signature had different immune activation and efficiency of immune checkpoints axis, leading to different benefit from immunotherapy.

### Construction and validation of a nomogram based on the B-lineage-associated risk signature

**Fig. 5:**
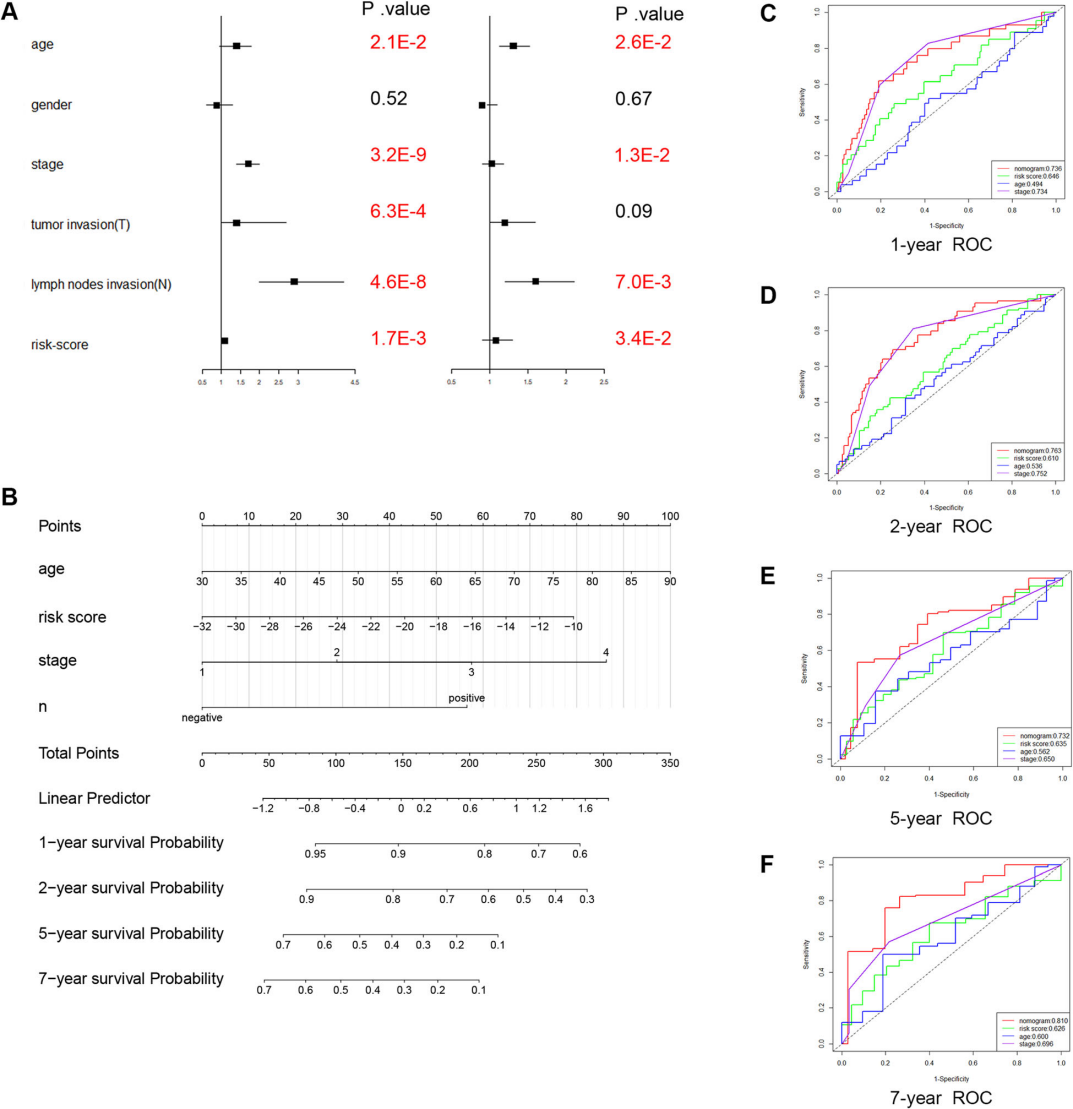
(**A**) Univariate cox regression analyses and multivariate cox regression analyses for lung adenocarcinoma patients in the TCGA cohort. Red indicates statistical significance (*P*-value< 0.05), and black indicates no statistical significance. (**B**) Nomogram for predicting the probability of 1-, 2-, 5- and 7-year OS for lung adenocarcinoma patients of TCGA cohort. Time-dependent ROC curve analyses of 4 factors, including age, TNM stage, the B lineage-associated risk signature and nomogram, 1-, 2-, 5- and 7-year in TCGA cohort

The predictive ability of the B-lineage-associated risk signature for prognosis was evaluated through univariate Cox regression analysis (*P* value < 0.05). Then, multivariate Cox regression was used to evaluate the B-lineage-associated risk signature score and several other clinical data, including TNM stage, lymph node invasion and age, as independent prognostic factors (*P* value < 0.05, Fig. 5A). A nomogram that integrated the B-lineage-associated risk signature and other independent clinical factors (lymphatic invasion, age, and TNM staging) was constructed to provide clinicians with a quantitative approach to predict the prognosis of patients with LUAD (Fig. 5B). Time-dependent ROC curve analysis was used to compare the predictive accuracy between the nomogram, B-lineage-associated risk signature, age, and TNM stage in 1, 2, 5, and 7 years (Fig. 5C–F). The nomogram model suggested higher prognostic accuracy for 1-, 2-, 5-, and 7-year OS with a larger AUC.

## Discussion

In our study, the MCP-counter algorithm was used to evaluate the immune cell infiltration of each sample in the TCGA-LUAD RNA-seq dataset. Among those microenvieonment cells, B-cell abundance significantly correlated with OS in patients with LUAD. A B-lineage-associated risk signature was constructed based on the TCGA cohort and validated in the meta-GEO cohort, which was significantly related to prognosis. The prognostic value of this signature was also independent of the known strong prognostic factors, such as sex, age, and tumor grade. In addition, this signature affected tumor immune-related pathways and immune cell infiltration in tumor tissues. Moreover, B-lineage-associated risk signature were positive correlate with several TILs marker, immune checkpoints and immunotherapy benefits. The molecular targets and several clinical factors were integrated into a new nomogram model with robust survival prediction, taking advantage of their complementary values.

LUAD is a malignant tumor with a high incidence worldwide. Early assessment of patient prognosis and effective immunotherapy biomarkers are very important. Traditional classification methods, including the TNM staging system, cannot cover the heterogeneity in molecular biology. Meanwhile, the research on the heterogeneity of the TME has become a hot issue in the field of tumor malignant progression, patient prognosis, and tumor immunotherapy [17–22]. Evaluating the prognosis of patients with LUAD from the perspective of molecular biology and TME is very meaningful for individualized diagnosis and treatment.

A large number of clinical trials have shown that the combination of immune checkpoint inhibitors and chemotherapy can significantly improve the progression-free survival of patients with advanced NSCLC compared with conventional chemotherapy alone [23, 24]. However, only part of patients can achieve a long-term, effective immune response from immunotherapy, and therefore a new immunotherapy strategy and research perspective is necessary [25]. As an important component of tumor-infiltrating immune cells, B cells may become a breakthrough in regulating immune-related therapeutic targets.

B cells can regulate immune response function through a variety of signaling pathways. Tumor-infiltrating B cells have been reported to exist in a variety of solid tumors [26]. B cells can inhibit the malignant progression of tumors by secreting immunoglobulins, promoting T-cell immune response, presenting tumor antigens, and directly killing tumor cells [27]. B cells and their related pathways work together to promote the aggregation, maturation, and maintenance of tertiary lymphatic structures (TLS) [28]. TLS is the lymphocyte aggregate formed in the chronic inflammatory response and similar in structure to the secondary lymphoid organs [29]. TLS is defined as a CD20+ B-cell follicle surrounded by a CD3+ T-cell aggregate of DC-LAMP+ mature dendritic cells [28, 30–32]. In many solid tumors including NSCLC, TLS is associated with improved prognosis and immune response [32, 33]. A total of 13 B-cell-associated transcripts were screened using bioinformatics methods, revealing that they had a strong interaction and co-regulation relationship. Hence, it was possible that they were from the same structure in the sample.

In the pathway enrichment analysis, we also found that a large number of RNA splicing-related pathways were enriched (Fig. S3A). Previous studies have shown that the RNA-binding protein hnRNPLL could splice and edit RNA in B cells, promoting the production of Ig and the loss of BCL6 expression, which indicated plasma cell maturation [34].. Meanwhile, previous studies revealed a total different RNA splicing status between B cell and plasma cell [35]. Those studies suggested us that, not only B cell infiltration, also B cell to matural plasma cell transforming, were different between two risk groups.

Our research still has some limitation and need further validation. Both our training cohort and the external validation cohort are carried out in a high-throughput public data queue. We need to use PCR to verify the effectiveness of B-lineage-associated risk signature in a larger real-world cohort. Otherwise, because it is difficult to obtain tissue samples from LUAD patients undergo immunotherapy, the correlation between B-lineage-associated risk signature and the benefit of immunotherapy is based on a small sample size public data cohort. We will verify B-lineage-associated risk signature in the large-scale immunotherapy cohort in the future.

In conclusion, the B-lineage-associated risk signature is a promising biomarker that divides patients into two subgroups with completely different clinical prognosis and immune status. It provides a view of the transcriptome level and TME to clarify the mechanism underlying different prognosis and efficacy of LUAD after immunotherapy.

## Supplementary Information

**Additional file 1: Supplementary Fig. S1.** (A) The landscape of the genes expression of RNA-seq data in the TCGA cohort. (B) The volcano plot reflected statistically differently expressed genes. (C) A heatmap reflected the cluster of high relative abundance of B lineage patients and low relative abundance B lineage patients in those differently expressed genes. (D) Tuning parameter (lambda) screening in the LASSO regression model. (E) The LASSO coefficient profiles of the common genes. (F) Selecting the optimal cutoff value in the B lineage-associated risk signature and divided patients into high-risk group and low-risk group.

**Additional file 2: Supplementary Fig. S2.** Stratification analysis. The Kaplan-Meier analysis of the B lineage-associated risk signature grouping according to patients with (A) male, (B) female, (C) young age (< 65 years), (D) elderly age (> 65 years), (E) early stage (TNM stage I), (F) advanced stage (TNM stage II, III, IV).

**Additional file 3: Supplementary Fig. S3.** Bar plots reflected the enriched biological processes, cellular components and molecular function of statistically differently expressed genes analysis between high relative abundance of B lineage patients and low relative abundance B lineage patients using (A) GO analysis and (B) KEGG analysis. (C) The gene expression profile interaction analysis of 13 risk signature-selected genes. (D) The correlationship of 13 risk signature-selected genes expression.

**Additional file 4: Supplementary Table S1.** Primers and RNA sequence used in this study.

**Additional file 5: Supplementary Table S2.** The differently expressed genes (Absolute value of LogFC> 1.5, *P* value< 0.05).

**Additional file 6: Supplementary Table S3.** The differently expressed genes (Absolute value of LogFC> 3, *P* value< 0.01).

**Additional file 7: Supplementary Table S4.** Coefficient of each gene in B lineage-associated risk signature.

**Additional file 8: Supplementary Table S5.** Clinical characteristics and PCR original data for 12 LUAD patients from Jiangsu Cancer Hospital.

## Data Availability

The data sets supporting the conclusion of this study are available from the “TCGAbiolinks” R package (Version 2.14.1; https://bioconductor.org/packages/release/bioc/html/TCGAbiolinks.html) and GEO database (https://www.ncbi.nlm.nih.gov/gds).
