# Supplementary figures and images for: A novel gene expression signature-based on B-cell proportion to predict prognosis of patients with lung adenocarcinoma
Source: BMC Cancer. 2021 Oct 12;21:1098. doi: 10.1186/s12885-021-08805-5 (PMC8513350; doi:10.1186/s12885-021-08805-5)

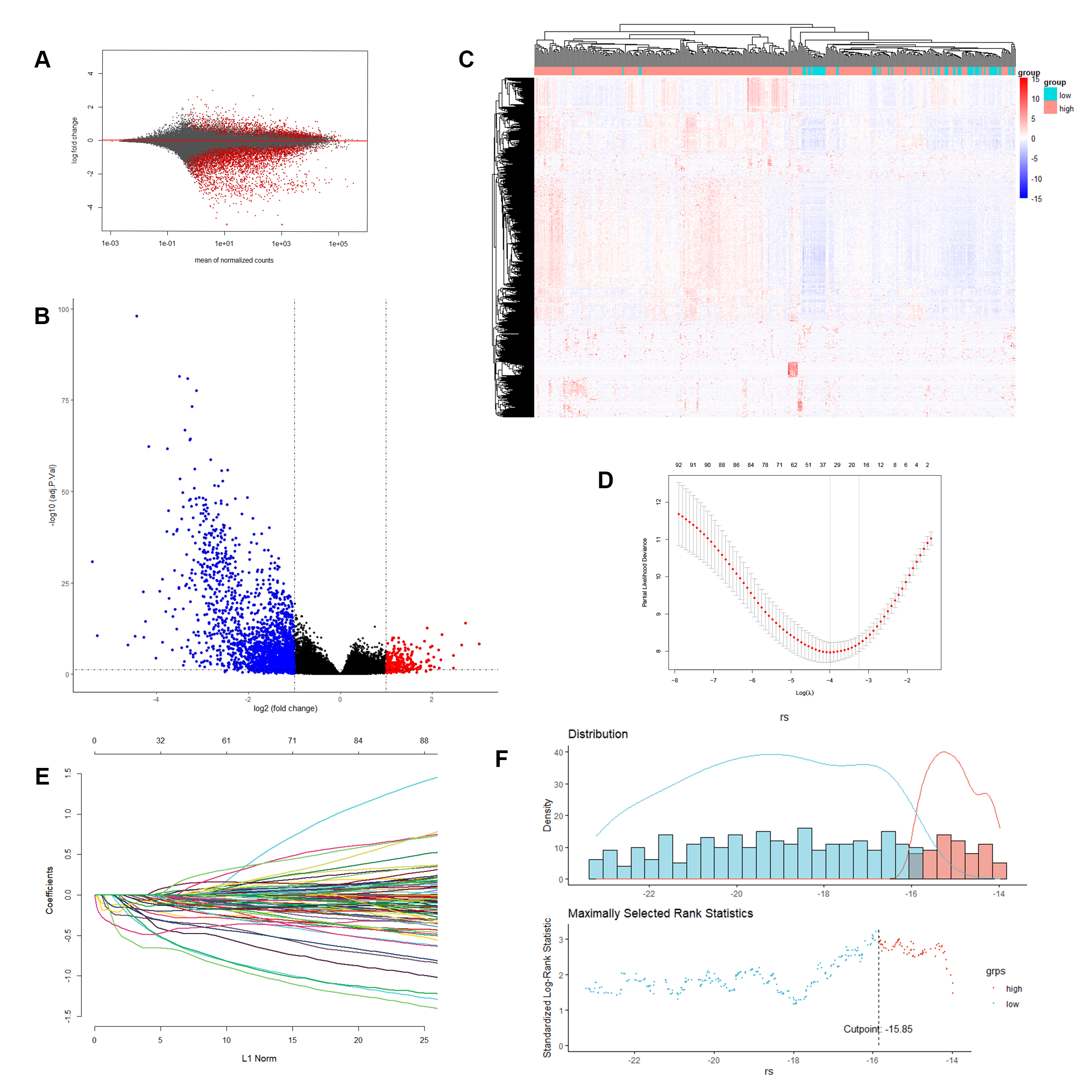

Supplement: Supplementary file 1 — Additional file 1: Supplementary Fig. S1. (A) The landscape of the genes expression of RNA-seq data in the TCGA cohort. (B) The volcano plot reflected statistically differently expressed genes. (C) A heatmap reflected the cluster of high relative abundance of B lineage patients and low relative abundance B lineage patients in those differently expressed genes. (D) Tuning parameter (lambda) screening in the LASSO regression model. (E) The LASSO coefficient profiles of the common genes. (F) Selecting the optimal cutoff value in the B lineage-associated risk signature and divided patients into high-risk group and low-risk group. [file 12885_2021_8805_MOESM1_ESM.jpg]

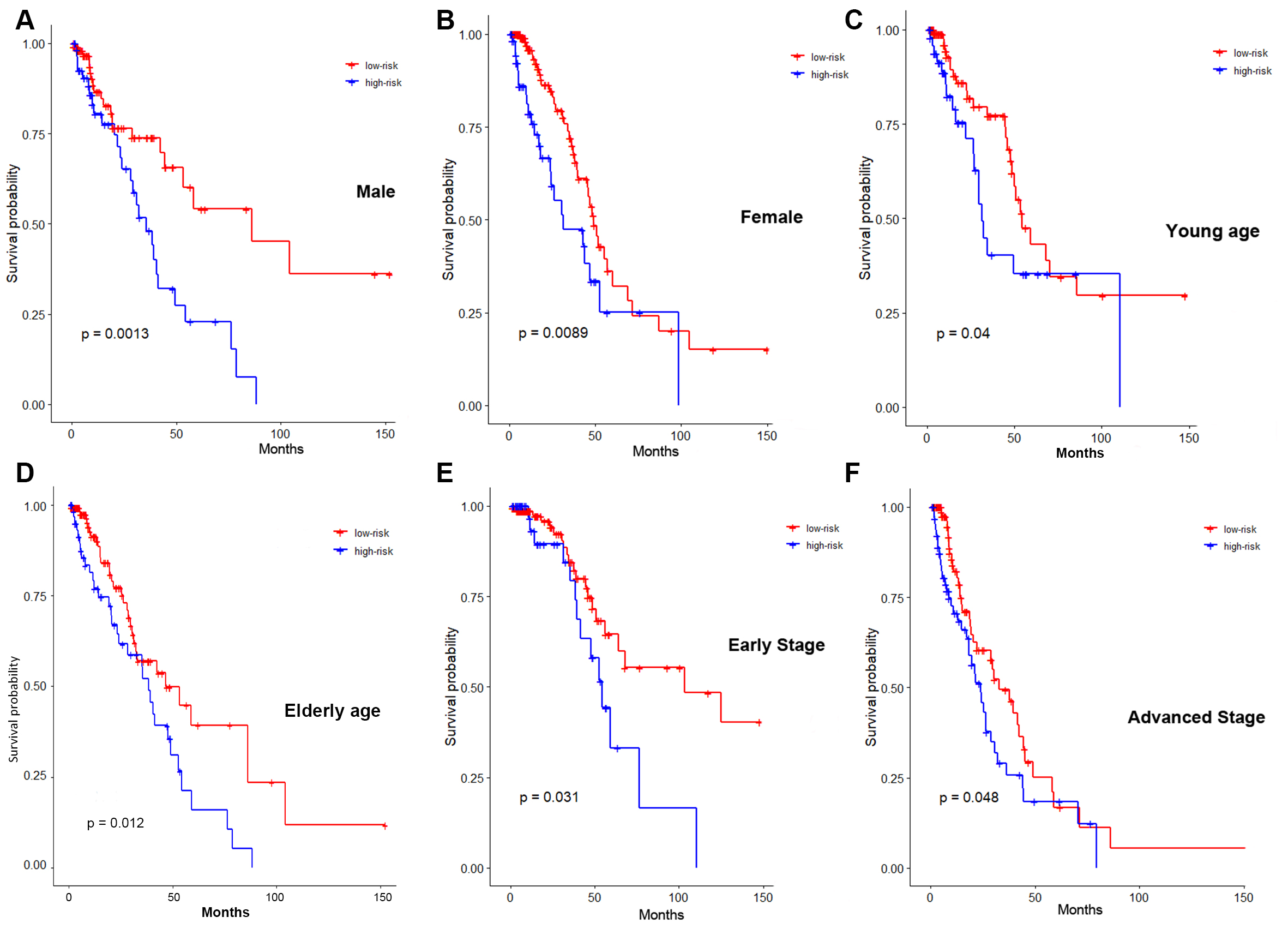

Supplement: Supplementary file 2 — Additional file 2: Supplementary Fig. S2. Stratification analysis. The Kaplan-Meier analysis of the B lineage-associated risk signature grouping according to patients with (A) male, (B) female, (C) young age (< 65 years), (D) elderly age (> 65 years), (E) early stage (TNM stage I), (F) advanced stage (TNM stage II, III, IV). [file 12885_2021_8805_MOESM2_ESM.jpg]

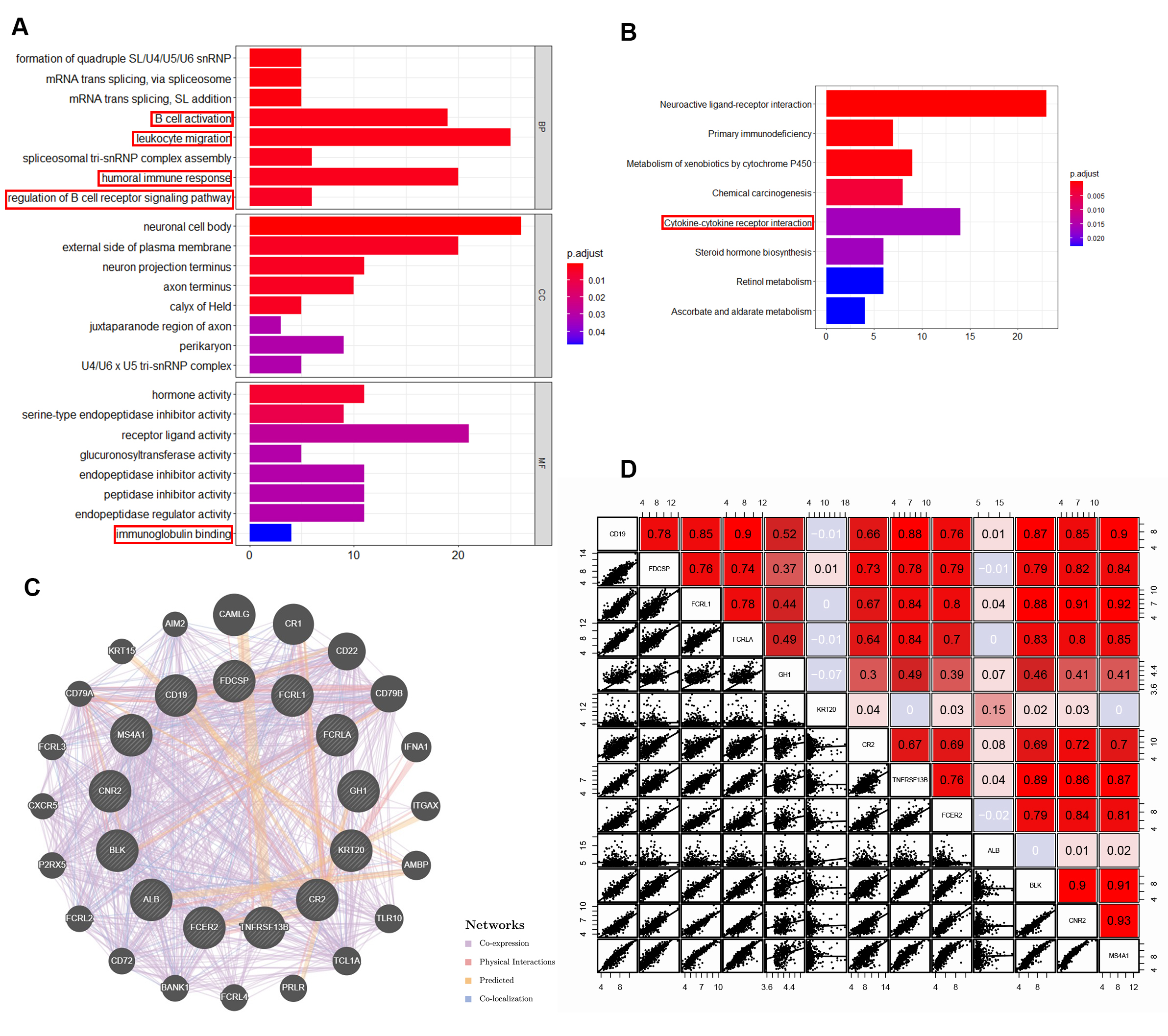

Supplement: Supplementary file 3 — Additional file 3: Supplementary Fig. S3. Bar plots reflected the enriched biological processes, cellular components and molecular function of statistically differently expressed genes analysis between high relative abundance of B lineage patients and low relative abundance B lineage patients using (A) GO analysis and (B) KEGG analysis. (C) The gene expression profile interaction analysis of 13 risk signature-selected genes. (D) The correlationship of 13 risk signature-selected genes expression. [file 12885_2021_8805_MOESM3_ESM.jpg]
