# Supplementary material for: A novel gene expression signature-based on B-cell proportion to predict prognosis of patients with lung adenocarcinoma
Source: BMC Cancer. 2021 Oct 12;21:1098. doi: 10.1186/s12885-021-08805-5 (PMC8513350; doi:10.1186/s12885-021-08805-5)
Supplement: Supplementary file 4 — Additional file 4: Supplementary Table S1. Primers and RNA sequence used in this study. [file 12885_2021_8805_MOESM4_ESM.docx]

**Table S1 Primers and RNA sequence used in this study**

**List of oligonucleotide sequences 5' --> 3'**

**primers for RT-PCR**

FDCSP-F AGTGGCTGTTGGTTTCCCAG

FDCSP-R GGCGAAATGGATATGGGTAAGG

FCER2-F GCGTGGGACTCAGATCGTG

FCER2-R GCTGTTTTAGACTCTGTGTGGTG

CNR2-F GGGTGACAGAGATAGCCAATGG

CNR2-R TGAACAGGTATGAGGGCTTCC

MS4A1-F CCGGCAGAGCCAATGAAAG

MS4A1-R CAGATGGGTGCATAGATCCCT

FCRL1-F GTGACCCTGACGTGTAAGATG

FCRL1-R TCGCACCAGTATGACCCTGT

BLK-F AGGAAAAGCCGATCAAAGAGAAG

BLK-R CCACCACGAAATGCTTGTCT

TNFRSF13B-F TTCAGACAACTCGGGAAGGTA

TNFRSF13B-R CATCGCGTGATCCTGGGAAG

CD19-F GGCCCGAGGAACCTCTAGT

CD19-R TAAGAAGGGTTTAAGCGGGGA

FCRLA-F CAGCCAAGCCAGTTTTTGAAG

FCRLA-R TCTGCCTTTTGTACCACGGTG

CR2-F GGTCCTCGGGATTTCTTGTGG

CR2-R GAACAACTGTACCTTATCACGGT

GH1-F ACCAGGAGTTTAGGCTGGAAG

GH1-R GCAGGAATGTCTCGACCTTGT

KRT20-F GGACGACACCCAGCGTTTAT

KRT20-R CGCTCCCATAGTTCACCGTG

ALB-F TGCAACTCTTCGTGAAACCTATG

ALB-R ACATCAACCTCTGGTCTCACC
