# Supplementary material for: A novel gene expression signature-based on B-cell proportion to predict prognosis of patients with lung adenocarcinoma
Source: BMC Cancer. 2021 Oct 12;21:1098. doi: 10.1186/s12885-021-08805-5 (PMC8513350; doi:10.1186/s12885-021-08805-5)
Supplement: Supplementary file 7 — Additional file 7: Supplementary Table S4. Coefficient of each gene in B lineage-associated risk signature. [file 12885_2021_8805_MOESM7_ESM.docx]

**Table S4 Coefficient of each gene in B lineage-associated risk signature**

**NCBI Gene Symbol Coefficient**

FCER2 -0.16936

CR2 -0.35238

FCRLA -0.16979

BLK -0.27845

MS4A1 -0.32492

FCRL1 -0.21464

ALB -0.01268

KRT20 -0.03807

CD19 -0.63325

FDCSP -0.17511

CNR2 -0.08366

GH1 -0.05848

TNFRSF13B -0.47873
